# Power Measurement Methods for Energy Efficient Applications

**DOI:** 10.3390/s130607786

**Published:** 2013-06-18

**Authors:** Guilherme Calandrini, Alfredo Gardel, Ignacio Bravo, Pedro Revenga, José L. Lázaro, F. Javier Toledo-Moreo

**Affiliations:** 1 Department of Electronics, University of Alcalá, Alcalá de Henares, Madrid 28871, Spain; E-Mails: alfredo@depeca.uah.es (A.G.); ibravo@depeca.uah.es (I.B.); revenga@depeca.uah.es (P.R.); lazaro@depeca.uah.es (J.L.L.); 2 Department of Electronics and Computer Technology, University of Politécnica Cartagena, ETSIT, Cartagena 30202, Spain; E-Mail: Javier.toledo@upct.es

**Keywords:** power measurement, energy consumption profiling, energy efficiency, instrumentation, power analysis

## Abstract

Energy consumption constraints on computing systems are more important than ever. Maintenance costs for high performance systems are limiting the applicability of processing devices with large dissipation power. New solutions are needed to increase both the computation capability and the power efficiency. Moreover, energy efficient applications should balance performance *vs.* consumption. Therefore power data of components are important. This work presents the most remarkable alternatives to measure the power consumption of different types of computing systems, describing the advantages and limitations of available power measurement systems. Finally, a methodology is proposed to select the right power consumption measurement system taking into account precision of the measure, scalability and controllability of the acquisition system.

## 1. Introduction

The performance of processing systems has found new constraints due to power consumption issues [1]. A common example is the maintenance cost of high performance computing (HPC) systems which can rapidly exceed the HPC acquisition price [2]. Therefore, novel solutions are needed to increase the computing performance (CP) while maintaining low energy consumption (EC). Nevertheless, the effort to enhance EC of computing units is not trivial and involves several areas of computing engineering, such as hardware design or software programming.

Although specific hardware design attains better results in both EC and CP, most users prefer to carry one flexible device rather than several separate devices [3], which has promoted the use of general-purpose solutions due to the low price of mass production. A well suitable example is the usage of graphic processing units (GPU) for general purpose computing [4].

An energy efficient application (EEA) should take into account hardware and software aspects in order to use just the required EC. Novel computing systems are heterogeneous, usually composed of multicore processors plus one coprocessor(s). Related to hardware, a review of Amdahl's law shows that heterogeneous architectures achieve better CP and EC [5] which has motivated several researches on asymmetric devices [6]. Though, hybrid architectures are not the panacea, further, large quantity of inefficient threads switching could lead to a waste of performance and EC. Other hardware alternatives have been proposed, for example the usage of low powered devices for HPC (LP-HPC) [7]. However, once that device was manufactured with their own hardware structure, the last solution available to improve EC is made by software *i.e.*, heterogeneous systems demand a power-efficient workload distribution [8]. Hence, the power consumption feedback for programmers can help to evaluate energy efficiency at software layers.

There are basically two main methodologies to collect EC: the first one is a hardware-based approach, this type of measurement obtains EC values through physical devices, measuring current and voltage in different test points. The second alternative makes use of power consumption models to estimate the EC. In addition, a third proposal could be used (a hybrid method formed by a mix of both). Nevertheless, the setup of an effective power model for a single device *i.e.*, a CPU is not a straightforward task; novel multicore and heterogeneous systems have turned this model even more complex. Although due to the inaccessibility of power lines/units inside of integrated circuits (IC), the estimation method is the unique alternative to establish internal EC in ICs. The constraints to measure performance on heterogeneous systems are presented in [9].

According to the previous arguments, a measurement system comes to help the development of EEA, which can be used to validate power models or analyze consumption. Real-time power consumption values help to develop new power management software techniques, such as power-aware job scheduling. Thereby, identification of power constraints can improve code programming of EEAs [10].

The EC constraints demand the introduction of useful measurement methods in current computing systems. In this paper, it is presented an up-to-date survey of different existing methods capable to measure efficiently the power consumption of generic computing systems. Besides, novel methods capable to retrieve power through model specific registers (MSR) included in new architectures are analyzed. Several measurement techniques have been tested, providing the reader relevant data to choose the right EC measurement for his application.

The next sections of this paper are organized as follows: Section 2 presents related works of classical power measurement techniques. The background on power measurement systems is shown in Section 3. Section 4 describes the available methods to measure computing power consumption, commenting generic implementations for each method. In Section 5, a computing system has been setup to execute EC profiling using several measurement methods. Finally, in Section 6 the main conclusions are presented.

## 2. Energy Consumption Measurement Methods on Computing Systems

Many researchers have analyzed how to retrieve values of EC from computing systems and make use of them in EEAs. Nevertheless, this issue is not standardized and there are different methods to retrieve the EC. In this section, relevant works related to power measurement and optimization solutions for EEAs are presented, describing the advantages and drawbacks of each system.

In [10] the power consumption for several GPU architectures is analyzed, concluding that algorithms can be classified in the two following categories according to its power consumption: data transfer intensive or computationally intensive. However the classification was made over time for GPU executions (transfer and execute kernel) with consumption measured from external power connectors obtaining rough EC values from computing platform, regardless of the communication interface.

Several works mention the importance of power optimization and advantages in use CPU power techniques while GPU performs some computation task, such as in [11]. Further, in [12] the EC of data transfer between CPU and GPU is analyzed, in particular, the CPU dynamic voltage and frequency scaling (DVFS) utilization, providing a methodology to find the optimal CPU frequency. The achieved DVFS values optimize the EC and CP of both CPU and GPU systems. It is worth to note that in this case the applied external measurement system fits the application interests.

Other solutions provide power models to estimate EC. The estimation can be performed through monitoring of the system usage at operating system level [13] or based in the hardware performance counters [14]. Similar methodology can also be applied for GPUs [15].

A common methodology to measure performance of processors is the usage of hardware performance counters, the method records the processor activity in the specific registers inside CPU (also called as MSR registers), to be accessed by user for performance monitoring. A similar software methodology may be applied to infer energy measurements. Due to the necessity of efficient power systems, the processors manufacturers have included an additional power management control unit (PCU) in hardware to enhance the power consumption, and protect the IC from thermal damage. Thus it is possible to manage power consumption of CPU through switching the compute units to different states via software. As a typical control system, the PCU requires an input to evaluate and determine the best power state for processing units, this input is built with an estimated power model [16], now made available for end users through specific registers.

Others researchers consider high level alternatives for EC profiling, such as: the insertion of known consumption behaviors as events to be correlated, and retrieves interested areas of consumption [17], although this alternative could not be applicable on multi task execution environments. Further, other solutions have been proposed such as: the *PowerPack*, a framework for EC profiling [18].

## 3. Measurement System Definitions

**Figure 1.**
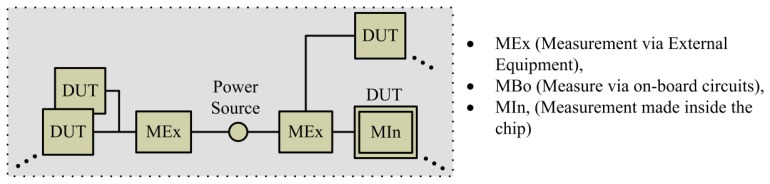
Measurement Systems Methods.

A measurement system has not a fixed structure. Therefore, let us introduce the main components for a generic measurement system. These systems are commonly composed of three basic components: the device under test (DUT), the measurement device (MD), and a power source. On computing systems, a DUT can be represented as a single processing unit, an entire processor (with multiple processing units and cache memories), a machine formed by a heterogeneous processing architecture (CPU + GPU) or a large system composed of several machines. These different granularities are commonly referred as the *measurement domains*. The MD is a generic device capable to measure power consumption, physically or making use of a power model, of a certain DUT. These basic units are presented in Figure 1 where MEx is a measurement device external to one or several DUT systems, and MIn represents a measurement available internally to a DUT component.

The *measurement scalability* of system can be defined by the possibility to attach more MDs or DUTs. Additional MDs can increase the *measurement domains*. Otherwise additional DUTs can bring collateral effects, due to requirement of MD calibration to cover the new range of power consumption [19].

The *measurement speed* defines how fast the measurement system can acquire data to represent any changes in consumption of the DUT.

The *synchronization accuracy* of a MD measures the feasibility to identify the instant power consumption for each part of the executed instructions. In [20], the author presents a generalized power delivery model and affirms that to implement a *cycle*-*accurate* measurement it is fundamental to take measure most closely possible to the source of consumption “*power sink*” due the low-pass filter effects of parasitic capacitance from delivery circuits.

The *measurement precision* reflects how the measured values resemble the real EC values, that is the uncertainty in the consumption, which may be fundamental if it is planned to make a power consumption quota distribution as in [20].

## 4. Energy Consumption Measurement Systems

In the following subsections different types of measurement systems capable to provide EC values of the computing units to the EEAs are presented.

### 4.1. External Measurements Systems

**Figure 2.**
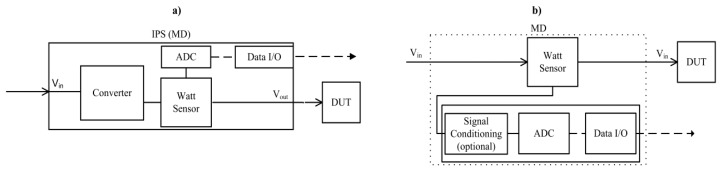
Measurement System Diagram (**a**) IPS based (**b**) *Ad-hoc* System.

In this type of MD an additional hardware capable to provide EC of one or more DUTs must be used. This is the case of an intelligent programmable power supply (IPS). An IPS is a power supply (see Figure 2(a)) with additional measurement and communication blocks. The I/O interfaces, such as USB/Ethernet are available in order to configure/read IPS parameters and collect EC values. The IPS collects periodically the current, voltage or wattage consumed by the DUT.

The main advantage of this alternative it is the facility to configure and start measurements. Although the scalability of the solution is possible, once the measurement system is setup the flexibility is low due to limitations of internal built-in circuit configuration. Besides, the accuracy could not fit the EEA needs, and the acquisition frequency may be too low for a correct EEA profiling. This type of MD may be of interest for quick tests and temporal analysis of EEAs; however the cost of IPS equipment which is usually expensive is a disadvantage.

Other external MD alternative could be the setup of a specific measurement system according to the EEA needs, for example: non-invasive, accuracy, cost, *etc.* making available some separate measures of the computing architecture. This specific hardware system could be deployed introducing additional physical sensors located in the input power lines of any potential DUT of the computing architecture. These solutions can provide a wide range of alternatives, such as the usage of current sensors based on hall-effect providing a non-intrusive solution (see Figure 2(b)).

The alternatives which use available sensors on the computing architecture are built on high level parts of software to retrieve values, without the insertion of additional hardware. This methodology is often denoted as “virtual probes”. The MD can be constructed using existing information of the system such as battery status [21] or thermal monitoring to obtain a measurement of E+C. However these solutions are not capable to provide accurate results due the time constraints of consumption drift.

In summary, external measurements may be introduced in different places of a computing architecture, *i.e.*, motherboard, PCI boards, *etc.* Next section is devoted to describe how new IC designs incorporate “measurement circuitry” to be able to obtain EC for a given portion of code, and more important to the manufacturer, limiting the voltage and frequency for the device if maximum thermal value is reached.

### 4.2. Internal Measurements

Due the inaccessibility of power lines from IC, recent trends motivate the usage of power models to estimate power consumption. The power modeling represent an accessible alternative to retrieve consumption from internal components of ICs which include several components all connected to a common external power lines. The integrated power model is an easy methodology to retrieve power through the software layers, *i.e.*,: the multicore processors. Such an example is the *Running Average Power Limit* (RAPL) interface present in the Intel Sandy Bridge microarchitecture [22]. The RAPL mechanism provides several measurement domains for this architecture, which are listed in Table 1 [23]. Nevertheless, the RAPL interface lacks in a fine-grain measurement for each core.

The measurement registers (MSRs) capture the EC of cores (PP0), integrated GPU if it is available (PP1) and internal memory consumption (DRAM). The data on MSRs are updated every millisecond, which provides a 1 kHz sampling of EC. Reading them at a frequency higher than 1 kHz may have significant overhead, thus this value of frequency sampling of EC represents a good balance. To isolate the consumption of a specific short code path, it is necessary to allow it to run continuously and read the MSR values synchronized, to reduce uncertainty to a minimum.

Different authors have analyzed the accuracy of RAPL registers to obtain EC of short code paths, such as [24]. In summary, with some minor considerations, the RAPL mechanism is good enough to be used for EC profiling.

## 5. Results

**Figure 3.**
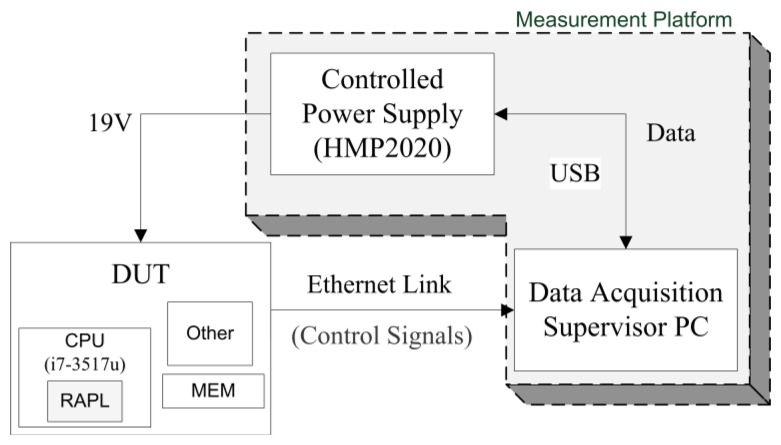
Measurement Testbench.

In this section, a testbench composed of several measurement methods previously described has been analyzed in order to show the different alternatives and extract conclusions about the use of EC measurement systems. To achieve relevant conclusions, the testbench shown in Figure 3 has been developed. The system is composed of a measurement platform and a device under test (DUT), in our case, an IvyBridge machine (Intel i7-3517u). This DUT has been chosen because this is a conventional CPU frequently used by many users in different tasks. Measurement platform will be connected with DUT by two ways: external power supply and ethernet channel to provide control signals and energy consumption data. Two measurement methods are used to evaluate. The first one is composed of an IPS as external measurement device while the second one makes use of the RAPL interface (internal measurement). RAPL is a novel approach in order to obtain an estimation of energy consumption by using the new hardware resources that manufacturers provide in new computing architectures. Thanks to both methods, a comparison between an external and internal proposal have been done. A supervisor PC is used to obtain the EC data from the IPS via USB and controls the code execution and RAPL-MSR reading inside the DUT. To avoid synchronization errors, the control signal of acquisition is sent 4 s before the start of processing and 4 s after the end of processing.

In order to evaluate a real workload for code execution, the FFTW library is used as a benchmark, a computing program based on Fast Fourier transform (FFT). This is typically used as a pattern to evaluate workloads on processing systems. Their internal complexity operations carry out many arithmetical operations providing a remarkable test to evaluate burden operations. In order to obtain an average value, the tests were carried repetitively 1,000 times over a FFT length of 1,048,567 and two types of executions were performed.

**Figure 4.**
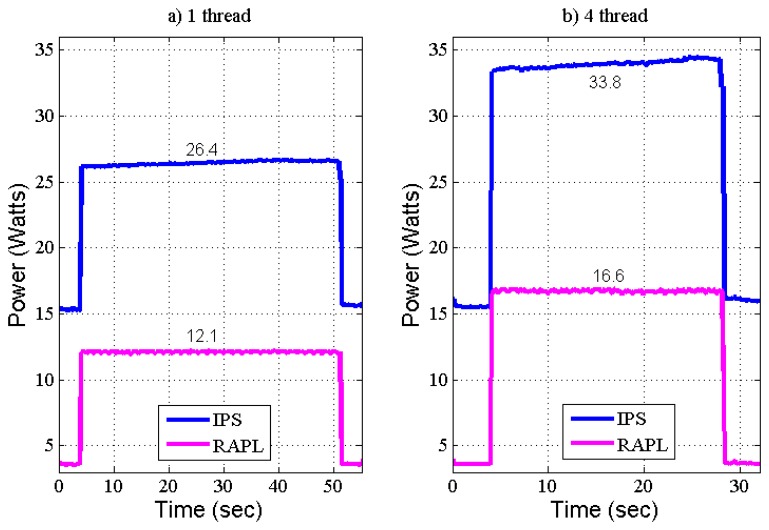
Testbench of Internal RAPL *vs.* External Measurements.

The first test uses 1 processing thread (Figure 4(a)). The execution of this workload starts at t = 4 s, performing the arithmetic computation in one core—one thread during 47 s. The second test uses 4 threads (Figure 4(b)) (maximum capacity of the multicore system under analysis), dual core with up to 2 threads running in parallel in each core. At maximum performance, this computing system process the workload from t = 4 s till t = 28 s, thus executing the FFTW testbench in 24 s, which represents a 2× speedup, from original performance. Concurrent accesses to memory reduce the theoretical 4× speedup executing 4 threads.

Energy consumption results of the FFT execution are shown in Figure 4. The blue lines present EC values obtained from the IPS, the magenta lines are the EC values taken from the RAPL interface at power plane PKG (see Table 1).

Thus, FFTW execution by one thread consumes 11.1 W apart from the 15.3 W of idle state. More processing (four threads) increases EC in 7.4 W (up to 33.8 W). The programmer and system designer could decide if the extra 7.4 W for a 2× speedup is well suited for current application. This additional energy consumption of 7.4 W, supposes a 21.9% of total consumption. This energy consumption data could be of great interest in energy-aware applications to decide about speedup the processing or reduce energy consumption.

The overhead of MSR reading and processing has been obtained making the external measurements with and without RAPL enabled, thus the overhead of internal measurements is 0.1 W. Due to RAPL method estimates just the CPU power consumption. The RAPL values follow the external measurement timing. The difference between both measurements represents the consumption of external peripheral devices, such as accesses to the main memory, chipset consumption, SSD, *etc.*

The results of average power consumption are summarized in Table 2. First seconds of the testbench maintains the computing system in idle mode. Idle EC gives for the complete system an average power of 15.3 W, which corresponds to 2.2 W for the processor device according to RAPL estimation. From the difference in power consumption of both data, a virtual measurement for the chipset, external memory, fan-ventilation, *etc.* is obtained (13.1 W).

In order to assess the power consumption between applications executing one thread or four threads, the next step is evaluate the results of both tests. Regarding the power consumption for one and four threads, executing one thread the power consumption increases in 11.1 W in comparison with idle state, 9.9 W corresponds to the processing device and 1.2 W to other system components. Far from being stable, the EC of system components raises when demanding more CP to the processing device. Thus, it is obtained from the made measurements that the performance increase executing four threads instead of one thread needs only 4.5 W more for the processing device (which represents an increase of 45% over the 9.9 W) and the rest of system components consumes additionally 2.9 W (which represents an increase of 242% over the 2.4 W executing only one-thread).

The CP obtained by four thread execution reduces to the half the processing time, but this demand of CP also increases in almost 2.5× times the EC from other system components. This consumption is mainly endorsed to the system memory. Therefore, for this kind of applications the memory optimization should be the main objective in order to increase power efficiency, but this is out of the scope of this paper.

## 6. Conclusions

The knowledge about EC on computing systems is more important than ever. Maintenance costs for high performance systems are limiting the applicability of processing devices with large dissipation power, and EEAs should balance performance *vs.* consumption. It is important to note that the methodologies presented here are not exclusive and can be used in parallel to build a specific measurement system.

In this paper, several EC measurement systems have been evaluated to retrieve power consumption in current computing systems. Main methodologies have been implemented in a real scenario. The obtained results demonstrate the importance of complementary methods of EC measurements. Thus, the data measurement provided by an IPS was extended by the usage of RAPL to distinguish the consumption between processing device and other peripheral devices.

A review of mentioned power measurement methods is presented in Table 3, which characterizes the methodologies according to the user interests. The lack of metrics of build process for a MSR method means that it is already included in the computing architecture. Further, some hardware devices available in the market for quick start measurement as can be the Watt's Up Pro (AC) power meter or the PowerMon (DC) have also been evaluated.

Thus, it is proposed a methodology to select the right power consumption measurement system taking into account precision of the measure, scalability and controllability of the acquisition system in order to manage the performance-consumption ratio efficiently. The measurement systems based on hardware sensors can be developed to measure the EC according to the end user needs. However the new processing solutions formed by several computing units integrated in a single die, difficult the measure process of internal components due to inaccessibility of separated power lines. Therefore, considering the integrated power models the best approach for new fused architectures, which can help programmers to profile their code and design EEAs.

The MSR approach to power measurement represents an innovative solution for EC profiling. Although this feature nowadays is hardware vendor dependent, the initiative to introduce energy counters will be standardized in the near future of computing systems. This approach will turn feasible to retrieve power consumption from complex computing systems with different processing units, network interfaces, memories, *etc.* at software level, enabling the development of power aware systems.

## Figures and Tables

**Table 1.** RAPL measurement domains.

**Power Planes**	**Domain**
**PKG**	The entire CPU package
**PP0**	CPU cores (including cache)
**PP1**	Uncore devices (*i.e.*, L3 cache and GPU)
**DRAM**	Memory

**Table 2.** Power consumption (Watts) analyses.

**Measurement Systems**	**Average Power Consumption (Watts)**					

	**Idle**	**One Thread**	**Diff 1th-Idle**	**Four Threads**	**Diff 4th-Idle**	**Diff 4th-1th**
**IPS**	15.3	26.4	11.1	33.8	18.3	7.4
**RAPL**	2.2	12.1	9.9	16.6	13	4.5
**Virtual Chipset Consumption Measurement**	13.1	14.3	2.4	17.2	5.3	2.9

**Table 3.** Power Measurement methodologies.

	**IPS (HMP2020)**	**Off-the-Shelf Sensor**		**RAPLMSR**

		Watts's UP Pro	ACS712 + μController	
**Easy to Build**	***	***	*	-
**Easy to Use**	***	**	**	***
**Scalability**	*	**	**	**
